# Modulation of Apoptotic Signaling by the Hepatitis B Virus X Protein

**DOI:** 10.3390/v4112945

**Published:** 2012-11-08

**Authors:** Siddhartha Rawat, Amy J. Clippinger, Michael J. Bouchard

**Affiliations:** 1 Graduate Program in Molecular and Cellular Biology and Genetics, Drexel University College of Medicine, Philadelphia, PA 19102, USA; Email: sr438@drexel.edu; 2 Department of Cancer Biology, Abramson Family Cancer Research Institute, School of Medicine, University of Pennsylvania Philadelphia, PA 19104, USA; Email: ajclippinger@gmail.com; 3 Department of Biochemistry and Molecular Biology, Drexel University College of Medicine, Philadelphia, PA 19102, USA

**Keywords:** hepatitis B virus, HBx protein, hepatocellular carcinoma, apoptosis

## Abstract

Worldwide, an estimated 350 million people are chronically infected with the Hepatitis B Virus (HBV); chronic infection with HBV is associated with the development of severe liver diseases including hepatitis and cirrhosis. Individuals who are chronically infected with HBV also have a significantly higher risk of developing hepatocellular carcinoma (HCC) than uninfected individuals. The HBV X protein (HBx) is a key regulatory HBV protein that is important for HBV replication, and likely plays a cofactor role in the development of HCC in chronically HBV-infected individuals. Although some of the functions of HBx that may contribute to the development of HCC have been characterized, many HBx activities, and their putative roles during the development of HBV-associated HCC, remain incompletely understood. HBx is a multifunctional protein that localizes to the cytoplasm, nucleus, and mitochondria of HBV‑infected hepatocytes. HBx regulates numerous cellular signal transduction pathways and transcription factors as well as cell cycle progression and apoptosis. In this review, we will summarize reports in which the impact of HBx expression on cellular apoptotic pathways has been analyzed. Although various effects of HBx on apoptotic pathways have been observed in different model systems, studies of HBx activities in biologically relevant hepatocyte systems have begun to clarify apoptotic effects of HBx and suggest mechanisms that could link HBx modulation of apoptotic pathways to the development of HBV-associated HCC.

## 1. Introduction

Epidemiological studies indicate that chronic infection with Hepatitis B Virus (HBV) is the leading cause for the development of hepatocellular carcinoma (HCC) [1]. According to the most recent data from Globocan 2008, which estimates the incidence, prevalence, and mortality of common cancers in the world, HCC is the fifth most common cancer in men and seventh most common in women [2]. Worldwide there were an estimated 694,000 deaths due to HCC in 2008 [2]. More than 50% of HCC cases worldwide and 70%–80% of HCC cases in HBV endemic areas are due to chronic HBV infection. Despite the availability of an HBV vaccine, approximately 350 million people are chronically infected with HBV worldwide, and HBV infection-related liver diseases remain a major global health problem [1].

**Figure 1:**
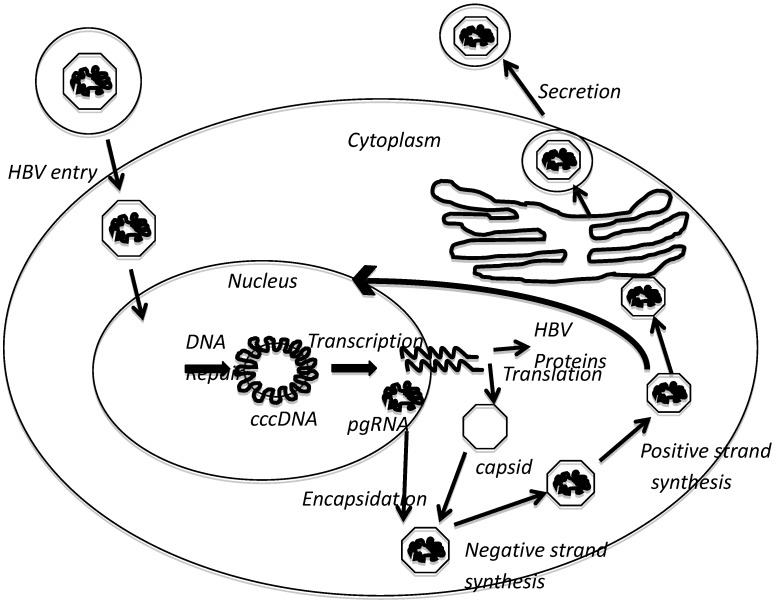
Hepatitis B Virus (HBV) life cycle. See text for details. pgRNA-pre-genomic RNA, cccDNA-covalently closed circular DNA, ER-endoplasmic reticulum.

HBV belongs to the *Hepadnaviridae* family of viruses. HBV has a highly compact genome of only 3.2 kilobases (kB) in length; this genome contains four overlapping open reading frames (ORFs) that encode the viral core protein (capsid), surface proteins (envelope), reverse transcriptase, and X protein (HBx). The viral genome is a partially double stranded, relaxed circular DNA that replicates by reverse transcription of an RNA intermediate. Hepatocytes are the predominant cells infected by HBV, and HBV host range and cell tropism are controlled by interactions between the virus and a cell surface receptor as well as hepatocyte-specific intracellular factors [3,4,5,6]. The cell surface receptor of HBV is not known, and the mechanism by which HBV enters hepatocytes is not clear. Although a recent report suggests that HBV enters hepatocytes through clathrin dependent endocytosis [7]. Following entry into hepatocytes, the HBV genome is delivered to the nucleus where the host-cell DNA repair machinery repairs the partially double-stranded DNA of the viral genome to form covalently closed circular DNA (cccDNA) (Figure 1). cccDNA is the template for transcription of viral mRNAs including the pre‑genomic RNA (pgRNA). Viral transcripts are then exported out of the nucleus into the cytoplasm and translated to form various viral proteins. pgRNA is packaged into viral capsids and is reverse transcribed by the virally encoded reverse transcriptase, which remains associated with the pgRNA, to generate the first DNA strand of the viral genome. The second complementary DNA strand of the viral genome is then completed to varying lengths, which gives rise to the partially double stranded DNA genome. Viral surface proteins envelop the capsids at the endoplasmic reticulum, and the packaged viral particles are secreted from cells using the secretory machinery of the host [8]. The envelope protein of HBV co-localizes with the proteins of multivesicular bodies in human hepatoma cells, and host multivesicular body functions are required for the release of enveloped HBV virions [9]. Some of the newly replicated genome in core particles is not secreted from cells but is instead delivered back to the nucleus to maintain a stable pool of nuclear cccDNA [8].

Although the association of chronic HBV infection with the development of HCC is well established, the exact molecular mechanisms or events that link HBV infection to HCC development are not completely defined. Three factors have been proposed to influence HCC development in a chronically HBV infected individual. These factors are: integration of viral DNA into the genome of the host cell, chronic inflammation due to the immune response of the host to the HBV infection, and HBV encoded proteins, such as HBx, that modulate cellular signal transduction pathways [10,11,12,13,14].

Integration of HBV DNA into the host cell genome has been observed in hepatocytes of chronically HBV-infected patients [15,16,17,18]. The integrated HBV DNA has been detected around the telomerase gene [19], cancer susceptibility regions, fragile sites [10,20], and the cyclin A gene [21]. Although these observations might suggest that there are preferred sites for HBV integration into the host genome, integration events were also reported to be random [22], and it remains unclear whether putative preferred integration sites reflect actual preferential integration or selective pressure for random integration events that are ultimately advantageous for cell survival and transformation. Some studies have suggested that the HBV HBx gene, and more specifically, truncated forms of the HBx gene, is preferentially integrated into the host cell DNA [23,24]. It is important to note, however, that studies have shown that an aberrant HBV replication intermediate, a double-stranded linear form of the viral genome, is a preferred form of the HBV genome that is integrated as compared to the circularized form of the genome [25]; this linear form of the viral genome has at its end the HBx gene. Therefore, the observed high frequency of integrated forms of the HBx gene may simply reflect preferential integration of a double-stranded linear form of the viral genome, or a double-stranded linear form of the HBV genome in which the ends of the linear HBV genome have been exposed to exonucleases prior to integration, rather than a preferential integration of the HBx gene. Overall, the relationship between integration of HBV DNA into the host genome and HCC development needs further investigation. For a description of the potential link between integration of HBV DNA into the host genome and the development of HCC, readers are referred to other more comprehensive reviews of this topic [10,11].

The second factor that is thought to have an important role in the development of HBV-associated HCC is the host immune response against the HBV infection, which is crucial for clearing the viral infection [4]. In chronically HBV infected individuals, immune mediated clearance of HBV-infected hepatocytes stimulates both clearance of HBV-infected hepatocytes and liver regeneration. During these cycles of hepatocyte clearance and regeneration, hepatocytes can accumulate mutations that ultimately contribute to transformation processes [14,26]. In addition, the immune response to an HBV infection causes infiltration of immune cells, release of inflammatory cytokines that can alter cell signaling pathways, and generation of reactive oxygen species (ROS) that can create a mutagenic environment leading to hepatocyte transformation [4,14]. Inflammation has been linked to many types of cancers, and changes in the microenvironment due to inflammation can influence angiogenesis and tumor cell metastasis [27]. Multiple pathways associated with inflammation, such as activation of the transcription factors Nuclear Factor kappa B (NF-κB) and Signal Transducer and Activator of Transcription 3 (STAT-3), cytokine production, and alterations of cellular signaling pathways can result in the development of HCC and are associated with an HBV infection [14,28]. Although the role of inflammation in the development of HBV-associated HCC is well established, a recent report of HCC development in HBV-transgenic mice suggests that HBV itself can be oncogenic in the absence of an inflammatory response [29]. For a comprehensive description of the role of the host immune response in HBV infection and HCC development readers are referred to recently published reviews [12,13]. 

The third factor that is thought to play a role in the development of HBV-associated HCC, and the subject of this review, is activities of the HBx protein in HBV-infected cells [14,30,31,32,33]. HBx is the major regulatory protein encoded by the HBV genome, and the results of numerous studies strongly suggest that HBx is involved in the development of HBV-associated HCC [14,34]. Although both mammalian and avian hepadnaviruses can establish chronic infections in their hosts, only chronic infections with the mammalian hepadnaviruses are associated with the development of liver cancer, and only the mammalian hepadnaviruses encode an X protein [6]. Avian hepadnaviruses either completely lack an X protein or encode a highly divergent form of this protein [35]. Studies conducted in cell culture and HBx-transgenic mice have also suggested a cofactor role of HBx in the development of HBV associated HCC (discussed in the next section). One controversial aspect of HBx biology that may be involved in the development of HBV-associated HCC is whether HBx modulates apoptotic pathways. HBx has been shown to stimulate, inhibit, or have no impact on apoptotic pathways depending on the conditions of a particular study [36,37,38,39,40,41]. In this review, we provide a brief overview of HBx biology, a review of the reported impact of HBx expression on apoptotic pathways, and a discussion of the potential role of HBx modulation of apoptosis in the development of HCC. Alterations of normal apoptotic processes have been linked to the development of HCC [42], suggesting that an understanding of how HBx impacts apoptotic pathways in hepatocytes may help identify molecular mechanisms that influence the development of HBV-associated HCC and potential contributions of HBx in this process. 

## 2. HBx Overview

The smallest open reading frame of mammalian hepadnavirus genomes encodes the 17 kilodaltons (kDa), multi-functional X protein. HBx, which is encoded by the human hepadnavirus HBV, is predominantly nuclear at low expression levels and mostly cytoplasmic at high expression levels; a fraction of cytosolic HBx co-localizes with mitochondria [36,43,44,45,46,47,48,49]. In HepG2 cells, a human hepatoblastoma cell line, and cultured primary rat hepatocytes, mitochondrial HBx is localized in the outer mitochondrial membrane, which is consistent with the reported interaction of HBx with the voltage-dependent anion channel (VDAC), a channel that spans the outer mitochondrial membrane and is thought to be a component of the mitochondrial permeability transition pore (MPTP) [43,44,50]. 

Although originally controversial due to the absence of any effect of HBx on HBV replication in human hepatoma Huh7 cells, there is now considerable evidence to support the notion that HBx is required for HBV replication [51]. HBx was shown to play a crucial role in HBV replication in cultured primary rat hepatocytes and HepG2 cells, and a recent report even suggested that HBx stimulates HBV replication in Huh7 cells [38,52,53,54,55,56]. The reason for the different outcomes in Huh7 cells is not known. While not absolutely required for HBV replication, HBx was also shown to enhance HBV replication in an HBV-transgenic mouse model [57]. In a more recent study in which a wild-type or HBx-deficient HBV genome was hydrodynamically injected into mice, the absence of HBx caused a 75% reduction in HBV replication in mouse hepatocytes and a 99% reduction in the level of circulating HBV [58]. In another study, a human hepatocyte chimeric mouse model in which mice hepatocytes are replaced with human hepatocytes was used to study the effect of HBx on HBV replication. The results of this study demonstrated that wild-type HBV infection resulted in measurable viremia after 2–7 weeks, whereas HBx-deficient HBV infection did not result in a measurable viremia up to 16 weeks after injection of the HBx-deficient HBV. However, HBV replication in HBx-deficient HBV-infected human hepatocytes in these chimeric mice was rescued by hydrodynamic tail vein injection of an HBx expression plasmid [59]. Finally, studies conducted by directly infecting cultured primary human hepatocytes with wild-type HBV or an HBx-deficient HBV also demonstrated the requirement for HBx in HBV replication [60]. Collectively, these studies provide evidence that HBx strongly stimulates and is likely required for HBV replication in normal hepatocytes. 

## 3. HBx and HCC

**Figure 2:**
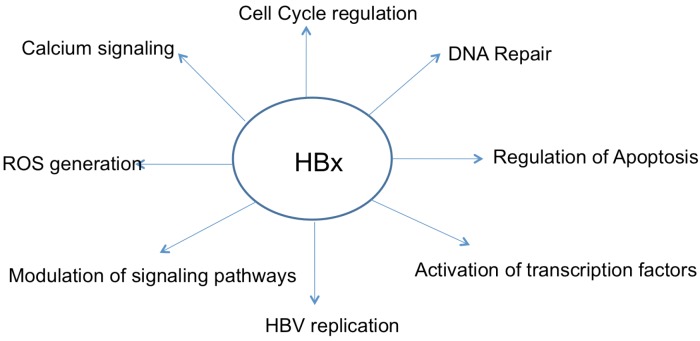
Various functions of HBx. HBx is known to modulate a number of cellular pathways as shown. Many of these pathways are interlinked and thus only a small number of upstream initiating events might be responsible for invoking a multifaceted response in hepatocytes upon HBx expression.

HBx can regulate cellular transcription, proliferation, DNA repair, apoptosis, and protein-degradation pathways, and these HBx activities have been invoked as potential mechanisms that link HBx expression and HBV replication to the development of HCC (Figure 2) [38,61,62,63,64,65,66]. HBx activities such as modulation of calcium signaling and regulation of the cell cycle have been shown to be important for HBV replication [55,65,67,68,69,70]. Whether HBx directly contributes to the development of HBV-associated HCC or functions as a co-factor in HCC development continues to be debated. In some HBx transgenic mouse models, HBx that was expressed in the absence of other HBV proteins directly facilitated the development of HCC [30,71]. In contrast, in other HBx-transgenic mouse models, HBx expression did not directly induce tumor formation but instead sensitized hepatocytes to carcinogen-induced HCC, suggesting a cofactor role of HBx in HCC development [72,73]. In a study conducted in HBV transgenic mice, HBV by itself could not induce HCC development but sensitized hepatocytes to diethylnitosamine (DEN) induced carcinogenesis [74]. In this model HBx was not essential for promoting DEN induced carcinogenesis but did enhance the efficiency of DEN induced carcinogenesis, possibly due to the effects of HBx on cellular signaling pathways or impairment of DNA repair [74]. More recently, the same group that earlier demonstrated that HBV transgenic mice were susceptible to DEN-induced carcinogenesis, but otherwise did not develop HCC up to 1 year of age, showed that these HBV transgenic mice when allowed to age for 2 years develop HCC in the absence of any carcinogenic treatment. Significantly, inflammation was not observed in the livers of the HBV-transgenic mice that developed HCC, suggesting that HBV on its own is carcinogenic [29]. Because a comparison was not made to HCC development in HBx-deficient HBV transgenic mice over the same time course, whether HBx directly influenced HCC development in the liver of these HBV-transgenic mice cannot be evaluated [29]. HBx has also been shown to decrease telomerase activity in HBx transgenic mice after a partial hepatectomy, suggesting that in the regenerating liver, HBx expression might lead to shortening of telomeres, which can cause chromosomal instability and contribute to malignant transformation [75]. In another study, HBx/c-myc bi-transgenic mice were created by crossing transgenic mice that expressed HBx in hepatocytes with transgenic mice that have liver-specific c-myc expression driven by woodchuck hepatitis virus (WHV). While HBx was not directly oncogenic in the original HBx transgenic mice, the HBx/c-myc bi-transgenic mice developed liver tumors, and the tumors in bitransgenic mice developed more rapidly as compared to WHV/c-myc transgenic mice, suggesting a cofactor role of HBx in c-myc induced tumor development [76]. Although the studies in the various HBx-transgenic mouse models have identified what appear to be mouse-strain specific responses to HBx expression, they none-the-less suggest that HBx can participate in hepatocyte transformation [37,71,73,77,78,79]. Overall, the results of most studies that have analyzed the impact of HBx expression on cellular signaling pathways that may contribute to hepatocyte transformation support a co-factor role for HBx in the development of HBV-associated HCC. A co-factor role for HBx in HBV-associated HCC is also more consistent with the biology of an HBV infection; although the entire hepatocyte population of the liver can be infected during a chronic HBV infection, transformation only occurs in a small number of hepatocytes and usually requires decades of a chronic HBV infection [3]. 

It is possible that multiple HBx activities contribute to the development of HBV-associated HCC, and many of the signaling pathways or cellular proteins that are regulated by HBx provide plausible mechanisms that could link HBx expression to the development of HBV-associated HCC [14,34]. While inflammation-mediated destruction of HBV-infected hepatocytes and subsequent liver regeneration contribute to the development of HBV-associated HCC, the results of studies in mice, established cell lines, and cultured primary hepatocytes suggest that activities associated with expression of HBx can regulate HBV replication, alter hepatocyte physiology, and modulate cell signaling pathways that may impact the development of HBV-associated HCC [14,34]. A critical evaluation of published studies supports the notion that HBx activities that influence HCC development are subtle. In fact, it is currently easier to study the role of HBx during HBV replication in animal models and tissue culture models than to assess the role of HBx in HCC development, which can take up to 30 years in a chronically HBV-infected individual [80]. During this time course of a chronic HBV infection, there can be fluctuations in the number of hepatocytes that are infected with HBV, the turnover of hepatocytes, the level of HBV replication, the expression of HBV proteins, and the immune response to the HBV infection [8,81,82], making it very difficult to directly assess the role of HBx in the development of HBV-associated HCC. With this as a consideration, one HBx activity that could directly impact the development of HBV-associated HCC is HBx modulation of apoptotic pathways [14,34]. De-regulation of normal hepatocyte apoptotic pathways is a well‑established mechanism that contributes to HCC development and progression [83,84]. Although discrepant observations have been published, there is considerable evidence that HBx can regulate apoptotic pathways, and this review will now focus on this HBx activity and its potential contribution to the development of HBV-associated HCC. 

## 4. Apoptosis

Apoptosis, or programmed cell death, is a highly regulated process that has a vital role in organ development and clearance of diseased or damaged cells [85]. Apoptosis can be initiated by extrinsic (originating outside a cell) or intrinsic (originating inside a cell) signaling pathways [86]. In the extrinsic pathway, binding of an extracellular ligand such as Fas to its receptor on the cell surface transmits a signal that activates an intracellular chain of events that cause cleavage and activation of procaspase-8 to form caspase-8. Caspases are cysteine-aspartate proteases that are essential for apoptosis [87]. Caspases can be initiator caspases such as caspase 2, 8, 9, and 10, which cleave the inactive form of procaspases, or they can be effector/executioner caspases such as caspases 3, 6, and 7, which cleave other protein substrates in cells. Caspase-8 is an initiator caspase that is activated in the extrinsic pathway of apoptosis and cleaves downstream caspases to eventually activate the executioner caspase, caspase-3 [87]. The intrinsic pathway is initiated by the release of cytochrome c from mitochondria along with other apoptosis-inducing factors such as second mitochondria-derived activator of apoptosis (Smac)/direct IAP binding protein with low pI (DIABLO) [86]. Cytochrome c forms an apoptosome complex with procaspase-9 and other apoptosis-regulatory proteins. Procaspase‑9 is cleaved to form caspase-9, which cleaves downstream caspases and activates caspase‑3. Caspase-3 cleaves various substrates such as Poly ADP-ribose polymerase (PARP), that ultimately causes the morphological and biochemical changes typically observed in apoptotic cells [85]. 

Depending upon the mechanism of apoptosis, cells can be classified as type I cells that require only caspase 8 mediated activation of executioner caspases or type II cells that require activation of caspase 8 as well as mitochondrial outer membrane permeabilization (MOMP) to release mitochondria-localized proteins that activate apoptotic pathways [86]. The crosstalk between extrinsic and intrinsic apoptotic pathways can be regulated by caspase 8-mediated cleavage and activation of BCL-2 homology 3 (BH3)-interacting domain death agonist (BID) [88,89]. BID in turn causes MOMP, which initiates the intrinsic pathway of apoptosis. The requirement for MOMP discriminates between type I and type II cells in death receptor mediated apoptosis [86]. Hepatocytes are type II cells and require the activation of BID followed by MOMP for complete activation of apoptosis [86]. This is demonstrated by the fact that anti-Fas ligand antibodies injected into mice cause hepatocellular apoptosis, but mice that are deficient in BID are protected when injected with the anti-Fas antibody and have no liver injury or activation of caspases 3 or 7, even though the initiator caspase 8 is activated [90]. Readers are referred to other comprehensive reviews for a detailed explanation of the role of MOMP in apoptosis [86,91,92].

## 5. HBx and Apoptosis

**Figure 3:**
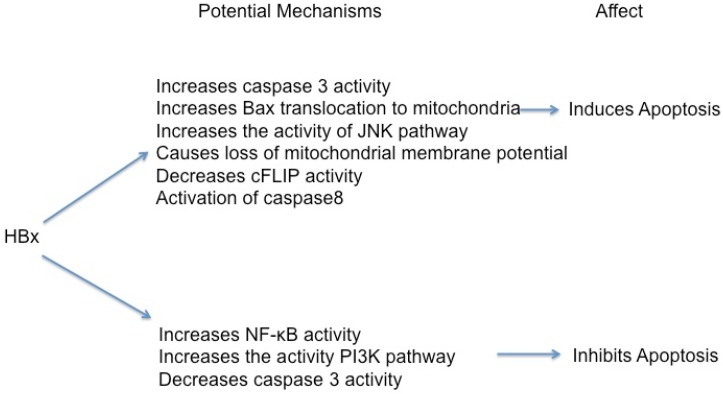
Potential mechanisms by which HBx induces or inhibits apoptosis in different cells. See text for details.

A large number of studies have assessed the impact of HBx expression on the regulation of apoptotic pathways, but the results of these studies have varied. HBx has been shown to induce [36,37,93,94,95,96,97,98,99,100,101,102,103,104,105,106,107], inhibit [38,39,108,109,110,111,112,113] or have no effect on apoptosis [40,41,114] in multiple cellular contexts (Figure 3). In addition, some studies have demonstrated that HBx does not directly induce apoptosis in certain cell types, but instead sensitizes these cells to pro-apoptotic stimuli [97,99,107]. It is likely that the seemingly discrepant apoptotic activities of HBx reflect differences in cell types used for these assays, whether studies were conducted in cells with transiently transfected *versus* constitutively expressed HBx, which may have facilitated cellular adaptation to HBx activities, and whether transgenic mouse hepatocytes expressed HBx at the time of analysis or had adapted to the continued expression of HBx during liver development. Some of the studies analyzing the effects of HBx on cellular apoptosis have also been conducted in non-hepatic cell lines, and since HBV does not infect these cells the relevance of the conclusions from such studies is uncertain. The potential influence of cellular context for HBx activities has been clearly demonstrated in studies using AML12 cells, a hepatocyte cell line derived from TGF-α transgenic mice [115]. During these studies in AML12 cells, stable HBx-expressing cell lines were generated in which HBx expression was controlled by a tetracycline responsive element. Two of these new AML12 derivative cell lines, 3pX-1 and 4pX-1, showed different extents of hepatocyte differentiation. Surprisingly, HBx sensitized de‑differentiated 4pX-1 cells, but not differentiated 3pX-1 cells to the activation of apoptotic pathways [99]. These observations, combined with reports that HBx can have different effects on apoptotic pathways in various experimental systems, supports the notion that the exact consequence of HBx expression on modulation of apoptotic pathways is likely influenced by the characteristics of the cells used for a particular study and demonstrate the importance of conducting experiments in systems that closely mimic physiologically relevant conditions. In the following sections, we will separately summarize studies that have demonstrated a pro-apoptotic effect of HBx and studies that have demonstrated an anti-apoptotic effect of HBx.

### 5.1. HBx Can Activate Apoptosis

HBx expressing Huh7 cells were shown to have increased terminal deoxynucleotidyl transferase dUTP nick end labeling (TUNEL), which detects DNA fragmentation, decreased orange and green color in 5,5',6,6'-tetrachloro-1,1',3,3'-tetraethylbenzimi-dazolylcarbocyanine iodide (JC-1)-loaded cells, which indicates a loss of mitochondrial membrane potential, and increased cytochrome c release from mitochondria into the cytosol, which demonstrates that MOMP has occurred, suggesting that HBx induces apoptosis in Huh7 cells [36]. The results of another study also showed an HBx-dependent increase in cytosolic cytochrome c levels, caspase-3-like activity, and nuclear fragmentation in HeLa cells and HepG2 cells [104]. In contrast, the results of a different study showed that HBx does not directly induce apoptosis in Chang and HepG2 cells but can sensitize these cells to Tumor necrosis factor-α (TNF-α) mediated apoptosis by activating the c-Jun amino-terminal kinase (JNK) pathway [97]. The results of this study further demonstrated that cytoplasmic localization of HBx is important for its ability to sensitize cells to TNF-α-mediated apoptosis; nuclear-localized HBx did not induce apoptosis [97]. The results of a different study showed that HBx sensitizes HepG2 cells and Chang cells to sub-apoptotic concentrations of TNF-α [93]. In this study, HepG2 cells were more resistant to HBx-mediated apoptosis than Chang cells, demonstrating the impact of cell-type-specific differences, and the increase in apoptosis was linked to the inhibition of cellular-FLICE like inhibitory protein (c-FLIP) activity and activation of caspase-8 by HBx [93]. As a cautionary note regarding studies in Chang cells, although this cell line was originally thought to be derived from human liver cells, isoenzyme analysis later revealed that at least some clones of this cell line may have been contaminated with HeLa cells [116]. HBx was also shown to induce cell death by localizing to mitochondria and causing loss of mitochondrial membrane potential in Huh7 and HepG2 cells [49]. The results of this study also demonstrated that the transcriptional activation function of HBx was not required for inducing apoptosis in these cells; a truncated HBx mutant that localized to mitochondria but had lost its transcriptional activation function was sufficient to cause mitochondrial membrane depolarization leading to apoptosis [49].

Another group examined the effect of HBx on apoptosis in Hep3B cells [95], a human hepatocellular carcinoma cell line that has stably integrated a copy of the HBV genome [117,118]. Hep3B cells were transiently transfected with a control or HBx expression plasmid because HBx mRNA or protein from the integrated HBV genome was undetectable. Transiently transfected HBx, with or without the addition of TNFα, induced apoptosis as measured by TUNEL analysis, and co‑expression of the anti-apoptotic protein B-cell lymphoma-extra large (Bcl-xL) abrogated this effect. HBx also promoted cytochrome c release, but this effect was only apparent in the presence of TNFα [95]. Interestingly, transfection with increasing amounts of the HBx-expressing plasmid showed a dose-dependent increase in apoptosis, suggesting that the level of HBx expression influences the percentage of cells undergoing apoptosis [95]. Transient transfection of HepG2 and MMDH3 cells, a mouse hepatocyte cell line, also demonstrated that HBx could induce apoptosis in the absence of any stimulus in these cells, and transfection of increasing amounts of the HBx-expressing plasmid revealed a dose-dependent increase in apoptosis in these cells [101]. 

Several groups have used cell lines stably expressing HBx to examine the effects of HBx on apoptosis. For example, one group monitored apoptosis in parental HepG2 cells and HepG2 cells stably expressing HBx [107]. In this study, HBx did not directly affect apoptosis; however, treatment of HBx-expressing cells with Vitamin K3 (VK3), which can induce apoptosis, sensitized cells to mitochondrial membrane depolarization and apoptosis as measured by DNA laddering and TUNEL analysis [107]. Apoptosis studies were also conducted in NIH 3T3 cells expressing HBx under the control of the dexamethasone-inducible mouse mammary tumor virus (MMTV) promoter. HBx induced apoptosis in serum-starved NIH 3T3 cells as measured by trypan blue exclusion analysis and by monitoring cell morphology [100]. Another group examined the effect of HBx expression in Chang cells stably expressing C-terminally human influenza hemagglutinin (HA)-tagged HBx under the control of a tetracycline-inducible promoter and demonstrated that HBx sensitized serum-starved Chang cells to the induction of apoptosis as measured by DNA laddering and nuclear condensation [105]. The effect of HBx on apoptosis in Chang cells stably-expressing N-terminally HA-tagged HBx expressed under the control of the simian virus 40 (SV40) promoter has also been examined [106]. Three Chang cell HBx-expressing cell lines were generated and were used for this study, each of which expressed a different level of HBx and showed different levels of HBx transcriptional activity. The Chang cell line with intermediate expression levels of HBx and highest level of transcriptional activity sensitized cells to apoptosis in the presence of staurosporine or cyclohexamide as measured trypan blue staining, cysteine protease protein-32 or caspase-3 (CPP32) cleavage, chromatin condensation, and a water soluble tetrazolium salt (WST-1) assay, which is based upon an enzymatic cleavage of tetrazolium salt by mitochondrial dehydrogenases that are present in viable cells. While the authors interpreted these results to suggest that HBx induces apoptosis, they also demonstrated that lower and higher expression levels of HBx did not sensitize cells to apoptosis. Additional studies in serum-starved HepG2 cells stably expressing HBx have demonstrated that HBx expression can increase DNA condensation, caspase-3 activity, cytochrome c release, and mitochondrial membrane depolarization in these cells [103]. The result of these studies also showed that HBx induces translocation of Bax to the mitochondria and suggested that HBx might interact with Bax [103]. Finally, in another study, inducible cell lines derived from HepG2 cells or human hepatoma HLF cells were created that had a loxP site-flanked, neomycin-coding region separating a CAG promoter and HBx coding sequence. When infected with a recombinant adenovirus expressing Cre recombinase, site-specific excision of neomycin coding region resulting in the formation of functional HBx expression unit controlled by the CAG promoter [96]. HBx expression increased the number of TUNEL-positive nuclei and the number of cells with sub-G1 DNA in HLF-derived cells. Additionally, HBx expression induced nuclear condensation in both HLF and HepG2 cells [96]. Overall, these studies suggest that HBx activation of apoptosis may be context-specific.

### 5.2. HBx Can Inhibit Apoptosis

Directly opposing a pro-apoptotic activity of HBx is evidence supporting a role for HBx in the inhibition of apoptosis [38,39,108,109,110,111,112,113,119]. HBx was shown to inhibit Fas-induced cell death in primary human hepatocytes as monitored by changes in cell morphology [39]. The results of one study suggested that HBx expression in HepG2 cells could partially protect HepG2 cells from Fas-mediated apoptosis; this effect of HBx was due to the activation of NF-κB by HBx [110]. The results of another study demonstrated that transiently transfected HBx inhibited apoptosis as measured by DNA fragmentation in Chang cells that were exposed to serum-deprivation, staurosporine, or etoposide treatment. In this study, the ability of HBx to inhibit apoptosis was due to the activation of the Phosphatidylinositol 3-kinase (PI3K) pathway; inhibition of the PI3K pathway blocked the anti‑apoptotic effect of HBx [108]. A similar study in Hep3B cells stably-transfected with HBx showed that HBx also inhibited TGF-β-induced DNA fragmentation through a PI3K-dependent pathway [109]. In another study, HBx was shown to activate the PI3K pathway and inhibit apoptosis by downregulating the expression of Phosphatase and tensin homolog (PTEN) in Chang cells [120]. HBx was also shown to inhibit apoptosis induced by growth factor depletion, TNF-α, or anti-Fas antibodies by inhibiting caspase-3 activity in rat fibroblasts and hepatoma cells [112]. The anti‑apoptotic property of HBx in these studies was attributed to two putative Kunitz domains present in the HBx sequence. *In vitro *studies using HepG2 cell extracts suggested that HBx might form a multi-protein complex containing the anti-apoptotic survivin protein to suppress caspase activation; however, this activity of HBx and its functional significance have not been verified *in vivo* [111]. The results of a recent study demonstrated that induction of apoptosis in HBV producing hepatocytes resulted in the release of non-enveloped HBV viral particles that had lower infectivity. HBx expression or HBV replication did not, in itself, result in sensitization of hepatocytes to apoptosis, and inhibition of apoptosis by HBV was required for the release of infectious HBV progeny, suggesting that the anti‑apoptotic function of HBx in normal hepatocytes may have an important role during HBV replication [121]. In another study conducted in HBV transgenic mice, it was shown that HBV replicates in these mice at levels comparable to the levels of HBV replication in chronically-HBV infected individuals; the hepatocytes of these transgenic mice did not show any HBV-associated cytopathic effect [122]. Finally, as discussed below, HBx was recently shown to inhibit apoptosis in cultured primary rat hepatocytes, which serve as a biologically relevant model of normal hepatocytes, the actual site of an authentic HBV infection. The anti-apoptotic activity of HBx was directly linked to HBx activation of NF-κB, and inhibition of NF-κB converted HBx from an anti-apoptotic factor to a pro-apoptotic factor [38].

### 5.3. HBx, NF-κB, and Apoptosis

NF-κB is overexpressed or constitutively activated in several cancers such as lung cancer, breast cancer, and pancreatic cancer [123,124,125]. NF-κB has also been shown to play an important role in development of liver cancer. When inhibitor of κ B kinase-β (IKK-β), which is required for NF-κB activation, was specifically deleted in mouse hepatocytes, these mice had increased hepatocarcinogenesis after DEN treatment [126]. Increased hepatocarcinogenesis was attributed to increased JNK pathway activation, hepatocyte death, and compensatory hepatocyte proliferation [126,127]. A study of patient liver tumor samples linked NF-κB activation to tumorigenesis in fibrolamellar hepatocellular carcinoma (FHCC), a rare form of HCC that is characterized by interspersed fibrous layers in the tumors [128]. In another clinical study, NF-κB activation was found in HCC patients, and there appeared to be a correlation between HBx expression and NF-κB activation in these patients. Moreover, NF-κB activation and urokinase type plasminogen activator upregulation were associated with more aggressive tumor behavior such as venous invasion, direct liver invasion, and absence of tumor encapsulation [129]. Thus, both activation and inhibition of NF-κB can result in the development of HCC through distinct mechanisms [130].

HBx activation of NF-κB and its relation to apoptosis has been extensively studied [131,132,133,134,135,136]; however, the mechanisms that underlie HBx activation of NF-κB remain unclear and may depend on the cellular context. For example, whether HBx activation of NF-κB functions through the Ras signaling pathway can differ in various cell types [131,132,137]. One group showed that dominant-negative mutants of focal adhesion kinase (FAK) or Ras inhibited HBx activation of NF-κB activity in HepG2 cells [132,137]. In contrast, another group showed that dominant-negative mutants of Ras and Raf did not inhibit HBx activation of NF-κB in HeLa cells [131]. The result of some studies have suggested that HBx activates NF-κB by inducing degradation of inhibitor of κ B (IκB); however, precisely how HBx affects IκB degradation is unclear [132,138]. IκB binds to NF-κB and prevents the translocation of NF-κB into the nucleus. IκB is phosphorylated by kinases such as IKK-β, releasing IκB from NF-κB, which allows NF-κB to translocate into the nucleus; phosphorylated IκB is degraded by the proteasome. Studies in Chang cells demonstrated that HBx activates NF-κB by inducing phosphorylation of IκB [132]. In contrast, the results of a different study in HeLa cells showed that HBx induced proteasomal degradation of IκB in the absence of IκB phosphorylation [138]. In addition, the results of some studies suggest that HBx activation of protein kinase C (PKC) is required for activation of NF-κB; however, PKC-dependence of HBx activation of NF-κB has also varied in different studies [135,139,140]. The discrepant observations of HBx activities that stimulate NF-κB provide additional evidence that HBx activities can be context dependent and demonstrate the importance of conducting studies in hepatocyte model systems that closely resemble physiologically relevant conditions. 

HBx regulation of NF-κB activity has been linked to HBx modulation of apoptosis in various experimental systems. For example, studies were conducted using a IκB-super repressor (SR) to examine the role of NF-κB during HBx modulation of apoptosis in NIH 3T3 cells; IκB-SR is a form of IκB that cannot be phosphorylated, remains constitutively bound to NF-κB, and blocks NF-κB activation [141]. HBx alone did not activate apoptosis; however, co-transfection of IκB-SR and HBx resulted in an increase in apoptosis as measured by trypan blue exclusion [102]. When HBx was expressed in Chang cells from a doxycycline-inducible promoter, apoptosis was only observed when the activity of NF-κB was inhibited [114]. In this study, HBx was shown to stimulate expression of chloramphenicol acetyl transferase (CAT) that was controlled by an NF-κB-dependent transcription promoter, and NF-κB inhibitors sulfasalazine and IκB-SR increased cell death in the presence of HBx [114]. The results of a more recent study demonstrated that HBx-dependent activation of NF-κB prevents apoptosis in cultured primary rat hepatocytes; however, when activation of NF-κB was blocked, HBx induced apoptosis through modulation of the mitochondrial permeability transition pore (MPTP) [38]. Importantly, in contrast to many previous studies in which only HBx activities were analyzed, the results of these studies in cultured primary rat hepatocytes were also confirmed when HBx was expressed in the context of HBV replication [38]. HBx was also shown to increase the expression of Methionine Adenosyltransferase 2A (MAT2A) in HepG2 cells, and increased expression of MAT2A was dependent on the binding of NF-κB and cAMP response element binding protein (CREB) to the transcription promoter of the MAT2A gene [142]. Importantly, overexpression of HBx or MAT2A inhibited apoptosis in HepG2 cells, and knockdown of MAT2A was pro-apoptotic [142]. Cumulatively, the results of published studies demonstrate that HBx can activate NF-κB in both primary hepatocytes and transformed or immortalized hepatocytes, although the specific mechanisms that underlie HBx activation NF-κB are not entirely understood. Moreover, HBx activates NF-κB in primary rat hepatocytes when HBx is expressed in context of HBV replication, and activation of NF‑κB is important for HBx-inhibition of hepatocyte apoptotic pathways. 

### 5.4. HBx, p53, and Apoptosis

p53 is a transcription factor that acts as a tumor suppressor, and loss or mutation of p53 is associated with many cancers [143]. p53 is activated by various factors including DNA damage, hypoxia, oncogene activation, and nutrient deprivation [143]. p53 can regulate cell cycle progression, apoptosis, DNA repair, and senescence [143], and mutations and polymorphisms of p53 have been associated with HCC development [144,145]. The results of some studies suggest that HBx can interact with p53 both *in vivo* and *in vitro*. A direct interaction between p53 and HBx was shown with *in vitro *translated HBx protein and purified p53 [146]. Immunoprecipitation experiments carried out in tumor and non-tumor tissues of ten HBV infected patients with HCC also revealed that p53 co‑precipitated with HBx [146]. Additional support for an interaction between HBx and p53 was provided by the results of a study that demonstrated that Glutathione-S transferase (GST)-p53 can interact with *in vitro*-translated HBx in a GST pull-down assay; the reciprocal interaction between GST-HBx and purified p53 was also shown [147]. The association between HBx and p53 was linked to a decrease in p53 DNA binding ability and inhibition of p53 transcription activity [147,148]. The results of another study suggested that HBx interacts with the carboxy-terminal domain of p53; this interaction inhibited p53-induced apoptosis in normal primary human fibroblasts [119]. The same group in a later report demonstrated that the distal C-terminal region of HBx (amino acids 111–154) was required for interaction with p53 and that the interaction between HBx and p53 sequestered p53 in the cytoplasm and abrogated p53-mediated apoptosis [113]. It is important to note, however, that many studies that demonstrated a direct interaction between HBx and p53 utilized over-expressed HBx and over-expressed p53, and whether endogenous p53 in hepatocytes interacts with HBx that is expressed from the HBV genome during an HBV infection is unknown.

The role of p53 in HBx-dependent regulation of apoptosis remains unclear, and there is evidence that HBx regulation of apoptotic pathways can be p53-dependent [100,149] and -independent [39,96,101]. In one study, HBx inhibited colony formation of mouse embryonic fibroblasts (MEFs) expressing normal p53 but did not inhibit colony formation in p53-deficient MEFs; these results were interpreted to suggest that HBx induces apoptosis in a p53-dependent manner [100]. Studies in HBx‑expressing AML12 cells demonstrated that HBx induced apoptosis in a de-differentiated HBx‑expressing cell line, 4pX-1, by activating the p38 mitogen activated protein kinase (p38MAPK) pathway [99]. HBx activation of p38MAPK stimulated p53, resulting in the transcription of the pro‑apoptotic genes Bcl-2 associated X protein (Bax), Fas, and Noxa and subsequent apoptosis [149]. Knockdown of p53 abrogated HBx-induced apoptosis in 4pX-1 cells, suggesting that HBx activates apoptosis through a p53-dependent mechanism in these cells. In contrast, there are also studies that demonstrate that HBx can induce apoptosis through a p53-independent mechanism. Studies in human hepatoma cells expressing HBx under the control of a Cre/loxP recombination system demonstrated that HBx induced apoptosis and sequestered inactive p53 in the cytoplasm in these cells, suggesting that HBx induced apoptosis through a p53-independent mechanism [96]. A comparison of the effect of HBx on apoptosis in p53-deficient DP-16 cells and p53-expressing DP-16 cells demonstrated that HBx inhibited Fas-mediated apoptosis regardless of the presence of p53, again suggesting that HBx can regulate apoptosis independent of p53 [39]. When p53-null mice were crossed with HBx-transgenic mice to examine the contribution of p53 in HBx-induced apoptosis, HBx stimulated apoptosis in p53‑expressing and p53-null hepatocytes, demonstrating that the effect of HBx on apoptosis was independent of p53 in these mice [101]. 

Whether p53 is required for HBV replication is unknown, but regulation of p53 by HBx could play an important role in HBV-associated HCC. The ability of HBx to sequester p53 and inhibit transactivation functions of p53 could inhibit cellular responses to damaged DNA such as control of cell cycle progression and apoptotic pathways and activation of DNA repair pathways, all of which can influence hepatocyte transformation and HCC development. This idea is supported by recent studies showing that HBx expression in hepatocytes and in HBx/c-myc bi-transgenic mice caused downregulation of ZNF198 and SUZ12 proteins, components of chromatin remodeling complexes. Downregulation of ZNF198 and SUZ12 was associated with increased HBV replication and resulted in a decrease in p53 levels and p53-mediated apoptosis, defective DNA repair, and increased oncogenic transformation [150,151]. Therefore, the putative interaction or crosstalk between HBx and p53, even if not necessary for the regulation of apoptotic signals, may still play a role in the development of HBV-associated HCC.

### 5.5. HBx, Reactive Oxygen Species, and Apoptosis

Highly active forms of oxygen such as hydrogen peroxide, superoxide anion, and hydroxyl radical are referred to as reactive oxygen species (ROS). ROS can be generated by multiple processes in cells including the electron transport chain in mitochondria, ionizing radiations, and through enzymes such as nicotinamide adenine dinucleotide phosphate (NADPH) oxidases [152,153,154]. ROS can be both good and bad for a cell depending upon ROS levels. When the levels of ROS are low in a cell, enzymes such as catalases and superoxide dismutases can neutralize ROS. At intermediate levels ROS can act as second messengers in signaling pathways, and at high levels, ROS can cause DNA damage and apoptosis, contributing to the pathogenesis of several diseases [155]. HBx has been shown to upregulate the levels of ROS in hepatocytes [49,56,133,156,157,158]. ROS generation due to the localization of HBx to mitochondria in Huh7 cells caused loss of mitochondrial membrane potential and cell death. Additionally, overexpression of the anti-apoptotic protein Bcl-xL or treatment with ROS scavengers prevented HBx-induced cell death [49]. HBx expression in HepG2 cells also resulted in downregulation of enzymes involved in the mitochondrial electron transport chain and increased mitochondrial ROS production [107]. HBx increased the expression levels of Cyclooxygenase-2 (COX-2) in Huh 7 cells; and this HBx activity was dependent on HBx elevation of ROS, supporting the role of HBx induced ROS as a second messenger. COX-2 is a key mediator of the inflammatory response and cirrhotic livers of HBV or HCV infected patients can have increased levels of COX-2 [159]. HBx has also been shown to enhance ROS production and sensitize HepG2 cells to H_2_O_2_ induced apoptosis. This pro-apoptotic effect of HBx in response to oxidative stress was attributed to the reduction of expression of Mcl-1, an anti-apoptotic protein [160]. Interestingly, HBx-expressing Chang cells that were treated with the chemotherapeutic agent adriamycin showed increased accumulation of ROS, which resulted in elevated HBx expression; this effect was abolished when the cells were treated with the antioxidants N-acetylcysteine and pyrrolidinedithiocarbamate. The results of this study suggested that ROS accumulation can also modulate intracellular levels of HBx; multiple reagents which can lead to ROS accumulation such as H_2_O_2,_ also showed a similar effect on HBx expression levels [156]. Regulation of HBx levels by ROS enhanced the effects of HBx on HBV replication and cell survival pathways. In contrast to a pro-apoptotic role of HBx induced ROS, the role of ROS as a second messenger in cell signaling was shown in Chang cells. Chang cells stably expressing HBx and primary hepatic tissue isolated from HBx transgenic mice had elevated levels of Forkhead box class O 4 (Foxo4) protein, which was dependent upon ROS production. Upregulation of Foxo4 was shown to inhibit oxidative stress induced apoptosis in Chang cells that stably expressed HBx [161]. 

**Figure 4:**
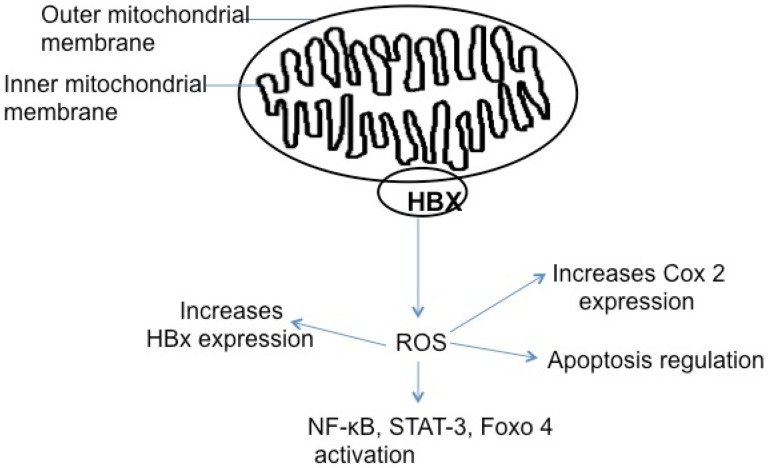
Localization of HBx to mitochondria results in ROS generation. ROS generation by mitochondria can lead to increased apoptosis due to loss of mitochondrial membrane potential. ROS has also been shown to play an important role in HBx induced activation of cell signaling pathways that have been shown to inhibit apoptosis.

Many transcription factors such as NF-κB, and signal transduction pathways such as the PI-3K and JNK pathways, that are activated by HBx are also regulated by ROS, and HBx mediated ROS elevation may underlie HBx regulation of these pathways. Specifically, studies in Huh7 cells demonstrated that HBx activation of NF-κB and STAT-3 is ROS-dependent, and quenching ROS using antioxidants abrogated the ability of HBx to induce NF-κB and STAT-3 DNA binding [133]. Since these transcription factors and signaling pathways can also affect apoptosis, HBx regulation of ROS might be a key player in HBx modulation of apoptotic pathways. Figure 4 summarizes the reported effects of HBx elevation of cellular ROS levels. HBx induced elevation of ROS has been shown to be both pro-apoptotic and anti-apoptotic. Cellular signaling events initiated by HBx-induced ROS accumulation could result in cell survival and induce hepatocyte proliferation. Excessive ROS can cause DNA damage or mutations leading to cellular transformation or apoptosis, which could initiate compensatory proliferation of hepatocytes. DNA damage due to increased ROS accumulation has been implicated in many cancers [162]. 

### 5.6. Effect of HBx on Apoptosis in HBx-Transgenic Mice

The results of apoptotic studies in various HBx-transgenic mouse models have been interpreted to suggest that HBx had no effect [40] or induced [37,98,101] apoptosis *in vivo*. These differences may reflect variations in the transgenic mouse strains, the promoters used to drive liver-specific HBx expression, the levels of HBx expression at the time of analysis, and the potential adaptation of mouse hepatocytes to continuous HBx expression [37,40,98,101]. For example, the transgenic mice used in some studies only expressed detectable HBx during the neonatal period, but not in adult mice [76,98,101]. In HBx-transgenic mice in which HBx was expressed under the control of the human α‑1‑antitrypsin regulatory region, no increase in hepatocyte apoptosis was observed [40]. Conversely, hepatocytes of transgenic mice with HBx expressed under the control of the human antithrombin III regulatory region had a moderate increase in TUNEL-positive hepatocytes as compared to control mice [101]. Additional experiments in which HBx-transgenic mice expressing HBx under the control of the human antithrombin III regulatory region were crossed with transgenic mice that over-express Bcl-2 demonstrated that HBx inhibited Bcl-2-mediated protection from Fas-induced apoptosis [98]. Apoptotic studies were also performed in transgenic mice expressing HBx under the control of its endogenous regulatory region; an increase in the number of TUNEL-positive cells was observed in these HBx-transgenic mice as compared to the control mice [37]. Finally, one study in HBV-transgenic mice demonstrated that HBV sensitized hepatocytes to TNFα-related apoptosis-inducing ligand (TRAIL)-induced apoptosis, although this was not directly linked to HBx expression in the HBV‑transgenic mice [94].

## 6. Conclusions and Perspective

The results of the numerous studies highlighted in this review provide compelling evidence that HBx can regulate cellular apoptotic pathways. The use of different cellular and transgenic mouse model systems, different levels of HBx expression, the presence or absence of growth factors, or whether the study was conducted in the context of HBV replication have sometimes generated contradictory observations. Collectively, the results of various studies suggest that HBx is capable of eliciting either an anti- or pro-apoptotic response and that this HBx activity is cell-context dependent. The cell-context specific effect of HBx presents the interesting possibility that the ability of HBx to induce or inhibit apoptosis may change during the course of an HBV infection as the liver is undergoing regeneration or as hepatocytes are undergoing transformation or responding to immune-cell-secreted cytokines, such as TNFα [163,164]. It is also possible that HBx has different effects in different microenvironments of the liver depending on the expression of key transcription factors, such as NF-κB, in that area. It is known that certain transcription factors are present at varying levels in different hepatic zones, and these local signals could affect HBx activities in different areas of the liver [165,166,167].

In studies in cultured primary rat hepatocytes, HBx was both pro- and anti-apoptotic, depending on the status of NF-κB activation. HBx activation of NF-κB prevented hepatocyte apoptosis, whereas in the absence of NF-κB activity, the ability of HBx to modulate the MPTP stimulated apoptosis [38]. While NF-κB inactivation inhibits carcinogenesis in some cell types [168], it can promote carcinogenesis in hepatocytes [126], and both inhibition and stimulation of apoptosis have been implicated in the development of HCC. Studies conducted in multidrug resistance 2 (Mdr2)-knockout mice demonstrated that NF-κB-dependent inhibition of apoptosis can promote liver tumor formation, and inhibition of NF-κB activity in these mice diminished tumor formation [169]. Conversely, repeated cycles of apoptosis and compensatory regeneration could eventually participate in the selection of hepatocytes that are resistant to pro-apoptotic signals and contribute to the development of HCC. For example, mice lacking IKKβ expression undergo more apoptosis and develop higher numbers of liver tumors than wild-type mice [126]. The results of one study conducted in HBx‑transgenic mouse demonstrated a delayed HCC development due to increased DNA synthesis and compensatory apoptosis [37]. HBx activation of NF-κB and inhibition of apoptosis could provide an environment that favors cell survival and tumor development. HBx-induced apoptosis and commensurate liver regeneration, which could occur in the context of diminished NF-kB activity in certain hepatic zones or as cells respond to various cytokines, could contribute to the eventual selection of hepatocytes that are resistant to pro-apoptotic signals, also resulting in the development of HCC. Cumulatively, the results of various studies suggest that during a chronic HBV infection of the liver, HBx could either induce or inhibit apoptosis, depending on the status of NF-κB activity and that both of these HBx effects could contribute to the development of HBV-associated HCC.

In summary, although it appears that HBx can regulate apoptosis, it is likely that in different cellular contexts the regulation of cellular signaling pathways by HBx is different. The development of HCC in a chronically HBV infected individual is a long process, and during this process the dominant cellular signaling pathways, microenvironment of the liver, and factors such as ROS might play an important role in defining the regulation of apoptosis by HBx. Different model systems used to study HBx regulation of apoptosis might provide clues regarding HBx apoptotic activities in hepatocytes as they progress through various stages of HCC development. The development of more human-relevant model systems, such as three-dimensional (3D) mini-liver systems composed of human liver cells will be critical in furthering our understanding of the role of HBV and apoptosis in the development of liver cancer [170,171,172] Although studies in primary hepatocytes support the notion that HBx is anti‑apoptotic and that this HBx activity is NF-κB dependent, seemingly discrepant observations in various system may be providing essential insights into cell-context specific activities of HBx that could also influence HBV replication, apoptosis, and HCC development. It is clear that additional studies are required to define mechanisms that underlie HBx regulation of apoptosis during a chronic HBV infection, how the liver environment influences this HBx activity, and how this HBx activity might change as hepatocytes undergo transformational processes. Importantly, a clear understanding of HBx regulation of apoptotic pathways during an HBV infection will help define mechanisms that regulate HBV replication and influence HCC development and may identify novel therapeutic strategies for preventing the development of HBV-associated HCC.
